# Certified Abstract Cost Analysis

**DOI:** 10.1007/978-3-030-71500-7_2

**Published:** 2021-02-24

**Authors:** Elvira Albert, Reiner Hähnle, Alicia Merayo, Dominic Steinhöfel

**Affiliations:** 8 Universidad Autónoma de Madrid, Madrid, Spain; 9 University of Twente, Enschede, The Netherlands; 10 Instituto de Tecnología del Conocimiento, Madrid, Spain; 11 Complutense University of Madrid, Madrid, Spain; 12 Technische Universität Darmstadt, Darmstadt, Germany; 13 CISPA Helmholtz Center for Information Security, Saarbrücken, Germany

## Abstract

A program containing placeholders for unspecified statements or expressions is called an abstract (or schematic) program. Placeholder symbols occur naturally in program transformation rules, as used in refactoring, compilation, optimization, or parallelization. We present a generalization of automated cost analysis that can handle abstract programs and, hence, can analyze the impact on the cost of program transformations. This kind of relational property requires provably precise cost bounds which are not always produced by cost analysis. Therefore, we certify by deductive verification that the inferred abstract cost bounds are correct and sufficiently precise. It is the first approach solving this problem. Both, abstract cost analysis and certification, are based on quantitative abstract execution (QAE) which in turn is a variation of abstract execution, a recently developed symbolic execution technique for abstract programs. To realize QAE the new concept of a cost invariant is introduced. QAE is implemented and runs fully automatically on a benchmark set consisting of representative optimization rules.

## 1 Introduction

We present a generalization of automated cost analysis that can handle programs containing placeholders for unspecified statements. Consider the program $Q \equiv$ "i =0; **while** (i < t) {P; i ++;}", where P is any statement not modifying i or t. We call P an *abstract statement*; a program like $Q$ containing abstract statements is called *abstract program*. The (exact or upper bound) cost of executing P is described by a function $\mathsf{ac}_\mathsf{P}(\overline{x})$ depending on the variables $\overline{x}$ occurring in P. We call this function the *abstract cost* of P. Assuming that executing any statement has unit cost and that $t \geq 0$, one can compute the (abstract) cost of $Q$ as $2 + t \cdot (\mathsf{ac}_\mathsf{P}(\overline{x}) + 2)$ depending on $\mathsf{ac}_\mathsf{P}$ and t. For any concrete instance of P, we can derive its concrete cost as usual and then obtain the concrete cost of $Q$ simply by instantiating $\mathsf{ac}_\mathsf{P}$. In this paper, we define and implement an abstract cost analysis to infer abstract cost bounds. Our implementation consists of an automatic abstract cost analysis tool and an automatic certifier for the correctness of inferred abstract bounds. Both steps are performed with an approach called *Quantitative Abstract Execution* (QAE).

Fine, but what is this good for? Abstract programs occur in program transformation rules used in compilation, optimization, parallelization, refactoring, etc.: Transformations are specified as rules over *program schemata* which are nothing but abstract programs. If we can perform cost analysis of abstract programs, we can *analyze the cost effect of program transformations.* Our approach is the *first method to analyze the cost impact of program transformations.*

*Automated Cost Analysis.* Cost analysis occupies an interesting middle ground between termination checking and full functional verification in the static program analysis portfolio. The main problem in functional verification is that one has to come up with a functional specification of the intended behavior, as well as with auxiliary specifications including loop invariants and contracts [21]. In contrast, termination is a generic property and it is sufficient to come up with a suitable term order or ranking function [6]. For many programs, termination analysis is vastly easier to automate than verification.[1]

Computation cost is not a generic property, but it is usually schematic: One fixes a class of cost functions (for example, polynomial) that can be handled. A cost analysis then must come up with parameters (degree, coefficients) that constitute a valid bound (lower, upper, exact) for all inputs of a given program with respect to a cost model (# of instructions, allocated memory, etc.). If this is performed bottom up with respect to a program's call graph, it is possible to *infer* a cost bound for the top-level function of a program. Such a cost expression is often *symbolic*, because it depends on the program's input parameters.

A central technique for inferring symbolic cost of a piece of code with high precision is *symbolic execution* (SE) [9, 25]. The main difficulty is to render SE of loops with symbolic bounds finite. This is achieved with *loop invariants* that generalize the behavior of a loop body: an invariant is valid at the loop head after arbitrarily many iterations. To infer sufficiently strong invariants automatically is generally an unsolved problem in functional verification, but much easier in the context of cost analysis, because invariants do not need to characterize functional behavior: it suffices that they permit to infer schematic cost expressions.

*Abstract Execution.* To infer the cost of program transformation *schemata* requires the capability of analyzing abstract programs. *This is not possible with standard SE*, because abstract statements have no operational semantics. One way to reason about abstract programs is to perform structural induction over the syntactic definition of statements and expressions whenever an abstract symbol is encountered. Structural induction is done in interactive theorem proving [7, 31] to verify, e.g., compilers. It is labor-intensive and not automatic. Instead, here we perform cost analysis of abstract programs via a recent generalization of SE called abstract execution (AE) [37,38]. The idea of AE is, quite simply, to symbolically execute a program containing abstract placeholder symbols for expressions and statements, just as if it were a concrete program. It might seem

[1] In theory, of course, proving termination is as difficult as functional verification. It is hard to imagine, for example, to find a termination argument for the Collatz function without a deep understanding of what it does. But automated termination checking works very well for many programs in practice.

counterintuitive that this is possible: after all, nothing is known about an abstract symbol. But this is not quite true: one can equip an abstract symbol with an *abstract* description of the behavior of its instances: a set of memory locations its behavior may depend on, commonly called *footprint* and a (possibly different) set of memory locations it can change, commonly called *frame* [21].

*Cost Invariants.* In automated cost analysis, one infers cost bounds often from loop invariants, ranking functions, and size relations computed during SE [3, 11, 16, 40]. For *abstract* programs, we need a more general concept, namely a loop invariant expressing a *valid abstract cost bound* at the beginning of any iteration (e.g., $2 + i * (\mathtt{ac}_\mathsf{P}(\overline{x}) + 2)$ for the program $Q$ above). We call this a *cost invariant*. This is an important technical innovation of this paper, increasing the modularity of cost analysis, because each loop can be verified and certified separately.

*Relational Cost Analysis.* AE allows specifying and verifying *relational* program properties [37], because one can express rule schemata. This extends to QAE and makes it possible, for the first time, to infer and to prove (automatically!), for example, the impact of program transformation on performance.

*Certification.* Cost annotations inferred by abstract cost analysis, i.e., cost invariants and abstract cost bounds, are automatically *certified* by a deductive verification system, extending the approach reported in [4] to abstract cost and abstract programs. This is possible because the specification (i.e., the cost bound) and the loop (cost) invariants are inferred by the cost analyzer—the verification system does not need to generate them.

To argue correctness of an abstract cost analysis is complex, because it must be valid for an infinite set of concrete programs. For this reason alone, it is useful to certify the abstract cost inferred for a given abstract program: during development of the abstract cost analysis reported here, several errors in abstract cost computation were detected—analysis of the failed verification attempt gave immediate feedback on the cause. We built a test suite of problems so that any change in the cost analyzer can be validated in the future.

Certification is crucial for the correctness of quantitative relational properties: The inferred cost invariants might not be precise enough to establish, e.g., that a program transformation does not increase cost for any possible program instance and run. This is only established at the certification stage, where relational properties are formally verified. *A relational setting requires provably precise cost bounds.* This feature is not offered by existing cost analysis methods.

## 2 QAE by Example

We introduce our approach and terminology informally by means of a motivating example: *Code Motion* [1] is a compiler optimization technique moving a statement not affected by a loop from the beginning of the loop body to before the loop. This code transformation should preserve behavior provided the loop is executed at least once, but can be expected to improve computation effort, i.e. *quantitative* properties of the program, such as execution time and memory

Program Before

```
int i = 0;
//@ loop_invariant i ≥ 0 && i ≤ t;
//@ cost_invariant
    i · (ac_P (t, w) + ac_Q (t, z) + 2) ;
//@ decreases t − i;
while (i < t) {
    //@ assignable x;
    //@ accessible t, w;
    //@ cost_footprint t, w;
    \abstract_statement P;
    //@ assignable y;
    //@ accessible i, t, y, z;
    //@ cost_footprint t, z;
    \abstract_statement Q;
    i++;
}
//@ assert \cost == 2 +
    t · (ac_P (t, w) + ac_Q (t, z) + 2) ;
```

Program After

```
int i = 0;
//@ assignable x;
//@ accessible t, w;
//@ cost_footprint t, w;
\abstract_statement P;
//@ loop_invariant i ≥ 0 && i ≤ t;
//@ cost_invariant
    i · (ac_Q (t, z) + 2) ;
//@ decreases t − i;
while (i < t) {
    //@ assignable y;
    //@ accessible i, t, y, z;
    //@ cost_footprint t, z;
    \abstract_statement Q;
    i++;
}
//@ assert \cost == 2 +
    ac_P (t, w) + t · (ac_Q (t, z) + 2) ;
```

Preconditions and Postconditions

Inputs: t, w, x, y, z  Precondition: $t > 0$  Postcondition: **cost_1** $\geq$ **cost_2**

Fig. 1: Motivating example on relational quantitative properties.

consumption: The moved code block is executed just once in the transformed context, leading to less instructions (less energy consumed) and, in case it allocates memory, less memory usage. In the following we subsume any quantitative aspect of a program under the term *cost* expressed in an unspecified *cost model* with the understanding that it can be instantiated to specific cost measures, such as number of instructions, number of allocated bytes, energy consumed, etc.

To formalize code motion as a transformation rule, we describe in- and output of the transformation *schematically*. Fig. 1 depicts such a schema in a language based on JAVA. An *Abstract Statement* (AS) with identifier *Id*, declared as "**abstract_statement** *Id*;", represents an arbitrary concrete statement. It is obviously unsafe to extract arbitrary, possibly non-invariant, code blocks from loops. For this reason, the AS P in question has a *specification* restricting the allowed behavior of its instances. For compatibility with JAVA we base our specification language on the *Java Modeling Language* (JML) [27]. Specifications are attached to code via structured comments that are marked as JML by an "@" symbol. JML keyword "**assignable**" defines the memory locations that may occur in the frame of an AS; similarly, "**accessible**" restricts the footprint. Fig. 1 contains further keywords explained below.

Input to QAE is the abstract program to analyze, including annotations (highlighted in light gray in Fig. 1) that express restrictions on the permitted instances of ASs. In addition to the frame and footprint, the *cost footprint* of an AS, denoted with the keyword "**cost_footprint**", is a subset of its footprint listing locations the cost expressions in AS instances may depend on. In Fig. 1, the cost footprint of AS Q excludes accessible variables i and y. Annotations highlighted in dark gray are *automatically inferred* by abstract cost analysis and are input for the certifier. As usual, loop invariants (keyword "**loop_invariant**") are needed to describe the behavior of loops with symbolic bounds. The loop invariant in Fig. 1 allows inferring the final value t of loop counter i after loop termination. To prove termination, the loop *variant* (keyword "**decreases**") is inferred.

So far, this is standard automated cost analysis [3]. The ability to *infer automatically* the remaining annotations represents our main contribution: Each AS P has an associated *abstract cost* function parametric in the locations of its footprint, represented by an abstract cost symbol $\mathsf{ac}_\mathsf{P}$. The symbol $\mathsf{ac}_\mathsf{P}\,(\mathsf{t},\mathsf{w})$ in the "**assert**" statement in Fig. 1 can be instantiated with any concrete function parametric in t, w being a valid cost bound for the instance of P. For example, for the instantiation "P $\equiv$ x=t+1;" the constant function $\mathsf{ac}_\mathsf{P}\,(\mathsf{t},\mathsf{w}) = 1$ is the correct *exact* cost, while $\mathsf{ac}_\mathsf{P}\,(\mathsf{t},\mathsf{w}) = \mathsf{t}$ with $\mathsf{t} \geq 1$ is a correct *upper bound* cost.

As pointed out in Sect. 1 we require *cost invariants* to capture the cost of each loop iteration. They are declared by the keyword "**cost_invariant**". To generate them, it is necessary to infer the *cost growth* of abstract programs that bounds the number of loop iterations executed so far. In Sect. 4 we describe automated inference of cost invariants including the generation of cost growth for all loops. Our technique is compositional and also works in the presence of nested loops.

The QAE framework can express and prove quantitative relational properties. The assertions in the last lines in Fig. 1 use the expression **cost** referring to the total accumulated cost of the program, i.e., the quantitative *postcondition*. We support quantitative relational postconditions such as **cost_1** $\geq$ **cost_2**, where **cost_1**, **cost_2** refer to the total cost of the original (on the left) and transformed (on the right) program, respectively. To prove relational properties, one must be able to deduce *exact* cost invariants for loops such that the comparison of the invariants allows concluding that the programs from which the invariants are obtained fulfill the proven relational property. Otherwise, over-approximation introduced by cost analysis could make the relation for the postconditions hold, while the relational property does not necessarily hold for the programs.

To obtain a formal account of QAE with correctness guarantees we require a mathematically rigorous semantic foundation of abstract cost. This is provided in the following section.

## 3 (Quantitative) Abstract Execution

Abstract Execution [37,38] extends symbolic execution by permitting abstract statements to occur in programs. Thus AE reasons about an *infinite* set of concrete programs. An abstract program contains at least one AS. The semantics of an AS is given by the set of concrete programs it represents, its set of *legal instances*. To simplify presentation, we only consider normally completing Java code as instances: an instance may not throw an exception, break from a loop, etc. Each AS has an *identifier* and a specification consisting of its frame and footprint. Semantically, instances of an AS with identifier P may at most write to memory locations specified in P's frame and may only read the values of locations in its footprint. All occurrences of an AS with the *same identifier* symbol have the same legal instances (possibly modulo renaming of variables, if variable names in frame and footprint specifications differ). For example, by

```
//@ assignable x,y;
//@ accessible y, z;
\abstract_statement P;
```

we declare an AS with identifier "P", which can be instantiated by programs that write at most to variables x and y, while only depending on variables y and z. The program "x=y; y=17;" is a legal instance of it, but not "x=y; y=w;", which accesses the value of variable w not contained in the footprint.

We use the shorthand $\mathsf{P}(\mathsf{x},\mathsf{y} :\approx \mathsf{y},\mathsf{z})$ for the AS declaration above. The left-hand side of ":≈" is the frame, the right-hand side the footprint. Abstract programs allow expressing a second-order property such as "all programs assigning at most x, y while reading at most y, z leave the value of i unchanged". In *Hoare triple* format (where $i_0$ is a fresh constant not occurring in P):

$$\{\mathsf{i} \doteq i_0\}\, \mathsf{P}(\mathsf{x},\mathsf{y} :\approx \mathsf{y},\mathsf{z});\, \{\mathsf{i} \doteq i_0\} \qquad (*)$$

### 3.1 Abstract Execution with Abstract Cost

We extend the AE framework [37,38] to QAE by adding *cost specifications* that extend the specification of an AS with an annotated *cost expression*. An abstract cost expression is a function whose value may depend on any memory location in the footprint of the AS it specifies. This location set is called the *cost footprint*, specified via the **cost_footprint** keyword (see Fig. 1), and must be a subset of the footprint of the specified AS. The cost footprint for the program in (∗) might be declared as "$\{z\}$". It implicitly declares the abstract function $\mathsf{ac}_\mathsf{P}\,(z)$ that could be instantiated to, say, quadratic cost "$z^2$".

**Definition 1 (Abstract Program).** *A pair $\mathcal{P} = (abstrStmts, p_{abstr})$ of a set of AS declarations abstrStmts $\neq \emptyset$ and a program fragment $p_{abstr}$ containing exactly those ASs is called* abstract program*. Each AS declaration in abstrStmts is a pair* $(\mathsf{P}(frame :\approx footprint), \mathsf{ac}_\mathsf{P}\,(costFootprint))$*, where* P *is an identifier; frame, footprint, and costFootprint $\subseteq$ footprint are location sets.*

*A concrete program fragment p is a* legal instance *of $\mathcal{P}$ if it arises from substituting concrete cost functions for all* $\mathsf{ac}_\mathsf{P}$ *in abstrStmts, and concrete statements for all* P *in abstrStmts, where (i) all ASs are instantiated legally, i.e., by statements respecting their frame, footprint, and cost function, and (ii) all ASs with the same identifier are instantiated with the same concrete program. The semantics $[\![\mathcal{P}]\!]$ consists of all its legal instances.*

The abstract program consisting of only AS P in (∗) with cost footprint "{z}" is formally defined as: $\big(\{(\mathsf{P}(\mathsf{x},\mathsf{y} :\approx \mathsf{y},\mathsf{z}), \mathsf{ac}_\mathsf{P}\,(\mathsf{z}))\}\,, \mathsf{P};\big)$. The program "$\mathsf{P}^0 \equiv$ i =0; **while** (i <z) {x = z; i ++;}" with cost function "$\mathsf{ac}_\mathsf{P}\,(\mathsf{z}) = 3 \cdot z + 2$" is a legal instance: it respects frame, footprint, and cost footprint, as well as the cost function, that (assuming $\mathsf{z} \geq 0$) can be obtained by static cost analysis of $\mathsf{P}^0$.

By encoding the semantics of abstract programs in a program logic [38, Sect. 4.2] one can statically verify whether an instance is legal. It may require auxiliary specifications (invariants, contracts) of the concrete code. The property is undecidable, but can be proven automatically in many cases, see [38] for a discussion. A first implementation of such a check is part of the REFINITY tool (see [36], also https://www.key-project.org/REFINITY/).

### 3.2 Cost of Abstract Programs

Finitely executing a concrete program $p$ starting in a state $s_0 = (p, \sigma_0)$ with an initial assignment $\sigma_0$ of $p$'s program variables results in a finite trace of the form $t \equiv s_0 \xrightarrow{c_1} \ldots \xrightarrow{c_n} s_n$. Each state $s_i = (p_i, \sigma_i)$ consists of a program counter $p_i$ (the remaining program to execute) and a store $\sigma_i$ (the current variable assignment); each transition $s_i \xrightarrow{c_{i+1}} s_{i+1}$ updates $s_i$ to $s_{i+1}$ according to the effect of executing command $c_{i+1}$ defined in the semantics of the programming language. A *complete* trace corresponds to a terminating execution, i.e., $s_n = (\epsilon, \sigma_n)$, where $\epsilon$ is the empty program and $\sigma_n$ the resulting final variable assignment.

The cost of a program can be computed based on execution traces. To allow arbitrary quantitative properties, we work on a generic *cost model* $\mathcal{M}$ that assigns cost values to programming language instructions. We will compute the cost of a trace $t$, denoted $\mathcal{M}(t)$, by summing up the costs of the executed instructions. A straightforward measure is the number of executed instructions $\mathcal{M}_{\mathsf{instr}}$: In this cost model, instructions like "x=1;", the evaluation of the loop guard, etc., all are assigned cost 1. For example, the cost of the complete trace of "**while** (i >0) i−−;" when started with an initial store assigning the value 3 to i is 7, because "i −−;" is executed three times and the guard is evaluated four times. This can be generalized to *symbolic* execution: Executing the same program with a *symbolic* store assigning to i a symbolic initial value $i_0 \geq 0$ produces traces of cost $2 \cdot i_0 + 1$. The cost of *abstract programs*, i.e., the generalization to QAE, is defined similarly: By generalizing not merely over all initial stores, but also over all concrete instances of the abstract program.

**Definition 2 (Abstract Program Cost).** *Let $\mathcal{M}$ be a cost model. Let an integer-valued expression $c_{\mathcal{P}}$ consist of scalar constants, program variables, and abstract cost symbols applied to constants and variables. Expression $c_{\mathcal{P}}$ is the* cost of an abstract program $\mathcal{P}$ w.r.t. $\mathcal{M}$ *if for all concrete stores $\sigma$ and instances $p \in [\![\mathcal{P}]\!]$ such that $p$ terminates with a complete trace $t$ of cost $\mathcal{M}(t)$ when executed in $\sigma$, $c_{\mathcal{P}}$ evaluates to $\mathcal{M}(t)$ when interpreting variables according to $\sigma$, and abstract cost functions according to the instantiation step leading to $p$. The instance of $c_{\mathcal{P}}$ using the concrete store $\sigma$ is denoted $c_{\mathcal{P}}(\sigma)$.*

*Example 1.* We test the cost assertion in the last lines of the left program in Fig. 1 by computing the cost of a trace obtained from a fixed initial store and instances of P, Q. We use the cost model $\mathcal{M}_{\mathsf{instr}}$ and an initial store that assigns 2 to t and 0 to all other variables. We instantiate P with "x=2∗t;" and Q with "y=i; y++;". Consequently, the abstract cost functions $\mathsf{ac_P}(\mathsf{t}, \mathsf{w})$ and $\mathsf{ac_Q}(\mathsf{t}, \mathsf{z})$ are instantiated with 1 and 2, respectively. Evaluating the postulated abstract program cost $2 + \mathsf{t} \cdot (2 + \mathsf{ac_P}(\mathsf{t}, \mathsf{w}) + \mathsf{ac_Q}(\mathsf{t}, \mathsf{z}))$ for the concrete store and AS instantiations results in $2+2\cdot(2+1+2) = 12$. Consequently, the execution trace should contain 12 transitions, which is the case.

### 3.3 Proving Quantitative Properties with QAE

There are two ways to realize QAE on top of the existing functional verification layer provided by the AE framework [37, 38]: (i) provide a "cost" extension to the program logic and calculus underlying AE; (ii) translate non-functional (cost) properties to functional ones. We opt for the second, as it is less prone to introduce soundness issues stemming from the addition of new concepts to the existing framework. It is also faster to realize and allows early testing.

The translation consists of three elements: (a) A global "ghost" variable "cost" (representing keyword "**cost**") for tracking accumulated cost; (b) explicit encoding of a chosen cost model by suitable ghost setter methods that update this variable; (c) functional loop invariants and method postconditions expressing cost invariants and cost postconditions.

Regarding item (c), we support three kinds of cost specification. These are, descending in the order of their strength: *exact*, *upper bound*, and *asymptotic* cost. At the analysis stage, it is usually impossible to determine the best match. For this reason, there is merely one **cost_invariant** keyword, not three. However, when translating cost to functional properties, a decision has to be made. A natural strategy is to start with the strongest kind of specification, then proceed towards the weaker ones when a proof fails.

An exact cost invariant has the shape "cost == *expr*", an upper bound on the invariant cost is specified by "cost <= *expr*"; asymptotic cost is expressed by the idiom "asymptotic(cost) <= asymptotic(*expr*)". The function "asymptotic" abstracts from constant symbols in the argument. For example, the (exact) cost postcondition of the abstract program on the right in Fig. 1 is:

$$\mathsf{cost}\ == 2 + \mathsf{ac_P}\,(\mathsf{t},\mathsf{w}) + \mathsf{t}\cdot(\mathsf{ac_Q}\,(\mathsf{t},\mathsf{z}) + 2) \qquad (\dagger)$$

Asymptotic cost would be expressed as $\mathsf{asymptotic}(\mathsf{cost})\ <= \mathsf{asymptotic}(2 + \mathsf{ac_P}\,(\mathsf{t},\mathsf{w}) + \mathsf{t}\cdot(\mathsf{ac_Q}\,(\mathsf{t},\mathsf{z}) + 2))$ where the right-hand side of the equation is equivalent to $\mathsf{asymptotic}(\mathsf{ac_P}\,(\mathsf{t},\mathsf{w}) + \mathsf{t}\cdot(\mathsf{ac_Q}\,(\mathsf{t},\mathsf{z})))$.

Listing 2 shows the result of translating the cost invariant in Fig. 1 to a functional loop invariant (highlighted lines), using cost model $\mathcal{M}_{\mathsf{instr}}$ in ghost setters and postconditions of AS ("**ensures**" clauses). ASs P, Q must include the ghost variable "cost" in their frame, because they update its value. The keyword **before** in the postcondition of an AS refers to the value a variable had just before executing the AS. In loops we use "inner" cost variables "iCost" tracking the cost inside the loop. When the loop terminates, we add the final value of "iCost" to "cost". After every evaluation of the guard of the loop, the cost is incremented accordingly. Using the translation in Listing 2 of the inferred annotations in Fig. 1, the AE system proves cost postcondition (†) automatically.

Apart from the translation of inferred quantitative annotations to functional AE specifications, we implemented the axiomatization of the asymptotic function and extended the AE system's *proof script* language. This made it possible to define a highly automated proof strategy for non-linear arithmetic problems generated by some cost analysis benchmarks.

## 4 Abstract Cost Analysis

Recall from Sect. 2 that for automatic cost certification we need to infer annotations for abstract cost invariants and cost postconditions. To achieve this, we

```
//@ ghost int cost = 0;
int i = 0;
//@ set cost = cost + 1;

//@ assignable x, cost;
//@ accessible t, w;
//@ ensures cost == \before(cost)
//@   + acP (t, w);
\abstract_statement P;

//@ ghost int iCost = 0;
//@ loop_invariant i ≥ 0 && i ≤ t
//@  && iCost == i · (acQ (t, z) + 2);
//@ decreases t − i;
while (i < t) {
    //@ set iCost = iCost + 1;
    //@ assignable y, cost;
    //@ accessible i, t, y, z;
    //@ ensures cost ==
    //@   \before(cost) + acQ (t, z);
    \abstract_statement Q;
    i++;
    //@ set iCost = iCost + 1;
}
//@ set cost = cost + 1;
//@ set cost = cost + iCost;
```

Listing 2: Translation of cost model and cost invariants to AE.

leverage a cost analysis framework for concrete programs to the abstract setting. The presentation is structured as follows: Sect. 4.1 defines the notion of an abstract cost relation system (ACRS) used in cost analysis for the abstract setting. Sect. 4.2 details how to generate automatically inductive cost invariants for abstract programs from ACRSs. Sect. 4.3 tells how to generate cost postconditions used to prove relational properties and required to handle nested loops.

### 4.1 Inference of Abstract Cost Relations

There are two main cost analysis approaches: those using recurrence equations in the style of Wegbreit [39], and those based on type systems [14, 24]. Our formalization is based on the first kind, but the main ideas for extending the framework to abstract programs would be also applicable to the second. The key issue when extending a recurrences-based framework to the abstract setting is the notion of *abstract cost relation* for loops which generalizes the concept of cost recurrence equations for a loop to an abstract setting. We start with notation for loops and technical details on assumed size relations.

```
while (G) {
  //@ accessible r_{1,1}, ..., r_{1,h_{r1}}
  //@ assignable w_{1,1}, ..., w_{1,h_{w1}}
  //@ cost_footprint c_{1,1}, ..., c_{1,h_{c1}}
  \abstract_statement A_1;
  non_abstract_statement N_1;
  ...
}
```

*Loops.* In our formalization we consider while-loops containing $n$ abstract statements and $m$ non-abstract statements. Non-abstract statements include any concrete instruction of the target language (arithmetic instructions, conditionals, method calls, ...). We assume loops $L$ have the general outline displayed on the right. Each abstract statement has a frame specification, abstract and non-abstract statements may appear in any order, either might be empty.

*Size relations.* We assume that for each loop sets of *size constraints* have been computed. These sets capture the size relation among the variables in the loop upon exit (called *base case*, denoted $\varphi_B$), and when moving from one iteration to the next (denoted $\varphi_I$). ASs are ignored by the size analysis. While this would be unsound in general, it will be correct under the requirements we impose in Def. 4 and with the handling of ASs in Def. 3. Size relations are available from any cost analyzer by means of a static analysis [13] that records the effect of concrete program statements on variables and propagates it through each loop iteration. In our examples, since we work on integer data, size analysis corresponds to a value analysis [10] tracking the value of the integer variables.[2]

*Example 2.* The size relations for the loop on the left in Fig. 1 are $\varphi_B = \{\mathsf{i} \geq \mathsf{t}\}$ and $\varphi_I = \{\mathsf{i} < \mathsf{t},\ \mathsf{i}' = \mathsf{i} + 1\}$. $\varphi_B$ is inferred from the loop guard and $\varphi_I$ from the guard and the increment of $i$ (primed variables refer to the value of the variable after the loop execution).

Based on pre-computed size relations, we define the cost of executing a loop by means of an *abstract cost relation system* (ACRS). This is a set of cost equations characterizing the abstract cost of executing a loop for any input with respect to a given cost model $\mathcal{M}$. Cost equations consist of a cost expression governed by size constraints containing applicability conditions for the equation (like $\mathsf{i} < \mathsf{t}$ in $\varphi_I$ above) and size relations between loop variables (like $\mathsf{i}' = \mathsf{i} + 1$ in $\varphi_I$).

**Definition 3 (Abstract Cost Relation System).** *Let $L$ be a loop as above with $n$ abstract and $m$ non-abstract statements. Let $\overline{x}$ be the set of variables accessed in $L$. Let $\varphi_I$, $\varphi_B$ be sound size relations for $L$, and $\mathcal{M}$ a cost model. The ACRS for $L$ is defined as the following set of cost equations:*

$$\begin{array}{ll} C(\overline{x}) = \mathtt{C_B} & ,\ \varphi_B \\ C(\overline{x}) = \sum_{j=1}^{n} \mathtt{ac_j}\left(c_{j,1}, \ldots, c_{j,h_{cj}}\right) + \sum_{i=1}^{m} \mathtt{C_{N_i}} + C(\overline{x}'), & \varphi_I \end{array}$$

*where:*

*(1) $\mathtt{C_B} \geq 0$ is the cost of exiting the loop (executing the base case) w.r.t. $\mathcal{M}$.*
*(2) Each $\mathtt{ac_j}(\cdot) \geq 0$ represents the abstract cost for the abstract statement $A_j$ in $L$ w.r.t. to $\mathcal{M}$. Each $\mathtt{ac_j}$ is parameterized with the variables in the cost footprint of the corresponding $A_j$, as it may depend on any of them.*
*(3) Each $\mathtt{C_{N_i}} \geq 0$ is the cost of the non-abstract statement $N_i$ w.r.t. to $\mathcal{M}$.*
*(4) $C$ is a recursive call.*
*(5) $\overline{x}'$ are variables $\overline{x}$ when renamed after executing the loop.*
*(6) The assignable variables $w_{j,*}$ in the $\mathtt{ac_j}$ get an unknown value in $\overline{x}'$ (denoted with "_" in the examples below).*

Ignoring the abstract statements, one can apply a complete algorithm for cost relation systems [6] to an ACRS to obtain automatically a *linear*[3] ranking function $f$ for loop $L$: $f$ is a linear, non-negative function over $\overline{x}$ that decreases strictly at every loop iteration. Function $f$ yields directly the "`//@` **decreases** $f$;" annotation required for QAE.

As in Sect. 3, the definition of ACRS assumes a generic cost model $\mathcal{M}$ and uses `C` to refer in a generic way to cost according to $\mathcal{M}$. For example, to infer the number of executed steps, `C` is set to 1 per instruction, while for memory usage `C` records the amount of memory allocated by an instruction.

[2] For complex data structures, one would need heap analyses [35] to infer size relations.
[3] There exist (more expensive) algorithms to obtain also polynomial ranking functions [5] but for the sake of efficiency we are not using them in our system.

*General Case of ACRS.* The definition of ACRS was simplified for presentation. The following generalizations, not requiring any new concept, are possible: (1) We assume an ACRS for a loop has only two equations, one for the base case (the guard G does not hold) and one for the iterative case (G holds). In general, there might be more than one equation for the base case, e.g., if the guard involves multiple conditions and the cost varies depending on the condition that holds on the exit. Similarly, there might be multiple equations in the iterative case, e.g., if the loop body contains conditional statements and each iteration has different cost depending on the taken branch. This issue is orthogonal to the extension to abstract cost. (2) A loop might contain method calls that in turn contain ASs. In absence of recursion, such calls can be inlined. For recursive methods, it is possible to compute the call graph and solve the equations in reverse topological order such that the abstract cost of the (inner) method calls is obtained first and then inserted into the surrounding equations. (3) The cost of code fragments not part of any loop (before, after, and in between loops) is defined as well by abstract cost equations accumulating the cost of all instructions these fragments include, just as for concrete programs. This aspect does not require changes to the framework for concrete programs, so we do not formalize it, but just illustrate it in the next example.

*Example 3.* The ACRSs of the programs in Fig. 1 are (left program above line, right program below):

$$
\begin{array}{rlr}
C_{\text{before}}(\mathsf{t},\mathsf{x},\mathsf{w},\mathsf{y},\mathsf{z}) &= c_{\text{before}} + C_{w_0}(\mathsf{i},\mathsf{t},\mathsf{x},\mathsf{w},\mathsf{y},\mathsf{z}), & \{\mathsf{i}=0\}\\
C_{w_0}(\mathsf{i},\mathsf{t},\mathsf{x},\mathsf{w},\mathsf{y},\mathsf{z}) &= c_{B_{w_0}}, & \{\mathsf{i}\geq \mathsf{t}\}\\
C_{w_0}(\mathsf{i},\mathsf{t},\mathsf{x},\mathsf{w},\mathsf{y},\mathsf{z}) &= c_{w_0} + \mathsf{ac}_{\mathsf{P}}(\mathsf{t},\mathsf{w}) + \mathsf{ac}_{\mathsf{Q}}(\mathsf{t},\mathsf{z}) + C_{w_0}(\mathsf{i}',\mathsf{t},\_,\mathsf{w},\_,\mathsf{z}), & \{\mathsf{i}'=\mathsf{i}+1,\mathsf{i}<\mathsf{t}\}\\
\hline
C_{\text{after}}(\mathsf{t},\mathsf{x},\mathsf{w},\mathsf{y},\mathsf{z}) &= c_{\text{after}} + \mathsf{ac}_{\mathsf{P}}(\mathsf{t},\mathsf{w}) + C_{w_1}(\mathsf{i},\mathsf{t},\_,\mathsf{w},\mathsf{y},\mathsf{z}), & \{\mathsf{i}=0\}\\
C_{w_1}(\mathsf{i},\mathsf{t},\mathsf{x},\mathsf{w},\mathsf{y},\mathsf{z}) &= c_{B_{w_1}}, & \{\mathsf{i}\geq \mathsf{t}\}\\
C_{w_1}(\mathsf{i},\mathsf{t},\mathsf{x},\mathsf{w},\mathsf{y},\mathsf{z}) &= c_{w_1} + \mathsf{ac}_{\mathsf{Q}}(\mathsf{t},\mathsf{z}) + C_{w_1}(\mathsf{i}',\mathsf{t},\mathsf{x},\mathsf{w},\_,\mathsf{z}), & \{\mathsf{i}'=\mathsf{i}+1,\mathsf{i}<\mathsf{t}\}
\end{array}
$$

Notation $c$ refers to the generic cost that can be instantiated to a chosen cost model $\mathcal{M}$. Cost equation $C_{\text{before}}$ for the first program is composed of the instructions appearing before the loop is $c_{\text{before}}$ plus the cost of executing the while loop $C_{w_0}$. The size constraint fixes the initial value of $\mathsf{i}$. Following Def. 3, there are two equations corresponding to the base case of the loop and executing one iteration, respectively. Observe that assignable variables in ASs have unknown values in the ACRS (according to item (6) in Def. 3). Program *after* has a similar structure. A ranking function for both loops is $\mathsf{t}-\mathsf{i}$ which is used to generate the annotation "`//@ decreases t−i;`" inserted just before each loop in Fig. 1.

To guarantee soundness of abstract cost analysis, it is mandatory that (i) no AS in the loop modifies any of the variables that influence loop cost, i.e., they do not *interfere with cost*, and (ii) the cost of the AS in the loop is independent of the variables modified in the loop. We call the latter ASs *cost neutral*. The first requirement is guaranteed by item (6) in Def. 3, because the value of assignable variables is "forgotten" in the equations. It is implemented, as usual in static analysis, by using a name generator for *fresh* variables. If cost depends on assignable variables in an AS, then the ACRS will not be solvable (i.e., the analysis returns "unbound cost"). The ACRS in the example contains "_" in equations that do not prevent solvability of the system nor its evaluation, because they do not interfere with cost. However, if we had "forgotten" a cost-relevant variable (such as t), we would be unable to solve or evaluate the equations: without knowing t the equation guard is not evaluable. Requirement (ii) is ensured by the following definition ensuring that variables in the cost footprint are not modified by other statements in the loop.

**Definition 4 (Cost neutral AS).** *Given a loop $L$, where*

- *$W(L)$ is the set of variables written by the non-abstract statements of $L$.*
- *$\texttt{Abstr}(L)$ is the set of all ASs in loop $L$.*
- *$\mathit{Frame}(\texttt{Abstr}(L))$ is the set of variables assigned by any AS $A \in \texttt{Abstr}(L)$.*
- *$\mathit{CostFootprint}(A)$ is the set of variables which the cost of an $A$ depends on.*

*$L$ is a loop with* cost neutral *ASs if, for all $A \in \texttt{Abstr}(L)$, it is the case that $(W(L) \cup \mathit{Frame}(\texttt{Abstr}(L))) \cap \mathit{CostFootprint}(A) = \emptyset$.*

The definition above constitutes a sufficient, but not necessary criterion that could be tightened by a more expensive analysis. For instance, our framework easily extends to allow conditions in the cost footprint that the concretizations of the AS must fulfill. In our example, the cost footprint might include condition $i' \geq i$, where $i'$ is the value of $i$ after executing the AS. This permits the abstract statement to modify $i$ provided it does not decrease its value. Thus, the AS is not cost neutral, but the upper bound remains sound. The formalization of this generalization is left to future work.

*Example 4.* It is easy to check that both loops in Fig. 1 have cost neutral ASs. On the left: $W(L) = \{\mathsf{i}\}$, $\mathit{Frame}(\{P, Q\}) = \{\mathsf{x}, \mathsf{y}\}$, $\mathit{CostFootprint}(P) = \{\mathsf{t}, \mathsf{w}\}$, and $\mathit{CostFootprint}(Q) = \{\mathsf{t}, \mathsf{z}\}$, so $(W(L) \cup \mathit{Frame}(\{P, Q\})) \cap \mathit{CostFootprint}(P) = \emptyset$, and $(W(L) \cup \mathit{Frame}(\{P, Q\})) \cap \mathit{CostFootprint}(Q) = \emptyset$. The program on the right is checked analogously.

Given a program $\mathcal{P}$ with variables $\overline{x}$ and ACRS with initial equation $C_{ini}(\overline{x})$. We denote by $\mathit{eval}(C_{ini}(\overline{x}), \sigma_0)$ the evaluation of the ACRS for a given initial assignment $\sigma_0$ of the variables. This is a standard evaluation of recurrence equations performed by instantiating the right-hand side of the equations with the values of the variables in $\sigma_0$ and checking the satisfiability of the size constraints (if the expression being checked or accumulated contains "_", the evaluation returns "unbound"). As usual, the process is repeated until an equation without calls is reached.

*Example 5.* Consider the ACRS of the left program in Fig. 1 with variables $(\mathsf{t}, \mathsf{x}, \mathsf{w}, \mathsf{y}, \mathsf{z})$, initial state $\sigma_0 = (2, 0, 0, 0, 0)$, and cost model $\mathcal{M}_{\text{inst}}$ (thus $c_{\text{before}}$, $c_{B_{w_0}}$ and $c_{w_0}$ take values 1, 1 and 2 respectively). The evaluation of the ACRS results in $\mathit{eval}(C_{ini}(\mathsf{t}, \mathsf{x}, \mathsf{w}, \mathsf{y}, \mathsf{z}), (2, 0, 0, 0, 0)) = 6 + 2 \cdot \mathsf{ac}_{\mathsf{P}}(2, 0) + 2 \cdot \mathsf{ac}_{\mathsf{Q}}(2, 0)$.

The following theorem states soundness of the ACRS obtained by applying Def. 3 provided that all loops satisfy Def. 4.

**Theorem 1 (Soundness of ACRS).** *Let $\mathcal{M}$ be a cost model and $\mathcal{P}$ an abstract program whose loops satisfy Def. 4. Let $c_{\mathcal{P}}$ be the abstract cost of $\mathcal{P}$ defined as in Definition 2. Let $C_{ini}$ be the initial equation for the ACRS obtained by Def. 3. For any initial state of the variables $\sigma_0 \in \mathbb{Z}^{n_m}$, it holds that $c_{\mathcal{P}}(\sigma_0) \leq eval(C_{ini}(\overline{x}), \sigma_0)$.*

### 4.2 From ACRS to Abstract Cost Invariants

Example 5 shows that ACRSs are evaluable for concrete instances. However, to enable automated QAE, we need to obtain from them *closed-form* cost invariants and postconditions, i.e., non-recursive expressions. We introduce the novel concept of *abstract cost invariant* (ACI) that enables automated, inductive proofs over cost in a deductive verification system. The crucial difference to (non-inductive) cost postconditions as inferred by existing cost analyzers is that ACIs can be proven inductively for each loop iteration. Hence, they integrate naturally into deductive verification systems that use loop invariants [21].

In contrast to ACIs, postconditions provide a bound for the cost *after* execution of the *whole* loop they refer to. Typically, a postcondition bound for a loop has the form $max\_iter * max\_cost + max\_base$, where $max\_iter$ is the maximal number of iterations of the loop, $max\_cost$ is the maximal cost of any loop iteration, and $max\_base$ is the maximal cost of executing the loop with no iterations. Instead, an ACI has the form $growth * max\_cost + max\_base$, where $growth$ counts how many times the loop has been executed and hence provides a bound after *each* loop iteration. The challenge is to design an automated technique that infers *growth*. We propose to obtain it from the ranking function:

**Definition 5 (Growth).** *Given a loop with ranking function $F = c + \sum_i a_i \cdot v_i$, where $c$ and $v_i$ are the constant and variable parts of the function, respectively, and $a_i$ are constant coefficients. If we denote with $v_i^0$ the initial value of variable $v_i$ before entering the loop, then $growth = \sum_i a_i \cdot \left(v_i^0 - v_i\right)$.*

*Example 6.* We look at four simple loops with ranking function *decreases* and the *growth* inferred automatically by applying Def. 5:

| | | | |
|---|---|---|---|
| **int** i = 0;<br>**while** (i < t)<br>i++; | **int** i = t;<br>**while** (i > 0)<br>i−−; | **int** i = 0;<br>**while** (i < t)<br>i += 2; | **int** i = t;<br>**while** (i > 0)<br>i −= 2; |
| *decreases* t − i<br>*growth* i | *decreases* i<br>*growth* t − i | *decreases* $\frac{t-i+1}{2}$<br>*growth* $\frac{i}{2}$ | *decreases* $\frac{i+1}{2}$<br>*growth* $\frac{t-i}{2}$ |

We can now define the concept of ACI that relies on abstract cost relations defined in Sect. 4.1 and growth as defined above.

**Definition 6 (Abstract Cost Invariant).** *Given an ACRS as in Def. 3 and its growth as in Def. 5, an* abstract cost invariant *is defined as follows:* $\mathtt{cinv}(\overline{x}) = \mathtt{C_B}^{\mathtt{max}} + growth \cdot \left(\sum_{j=1}^{n} \mathtt{ac_j}\left(c_{j,1}, \ldots, c_{j,h_{cj}}\right) + \sum_{i=1}^{m} \mathtt{C_{N_i}}^{\mathtt{max}}\right)$ *where* $\mathtt{C_B}^{\mathtt{max}}$ *stands for the maximal value that the expression* $\mathtt{C_B}$ *can take under the constraints* $\varphi_B$*, and* $\mathtt{C_{N_i}}^{\mathtt{max}}$ *the maximal value of* $\mathtt{C_{N_i}}$ *under* $\varphi_I$*. We generate the annotation "//@* **cost_invariant** $\mathtt{cinv}(\overline{x})$*;".*

To obtain the maximal cost of a cost expression under a set of constraints, we use existing maximization procedures [5].

From Def. 6 we obtain ACIs as closed-form abstract cost expressions of the form $\texttt{abexpr} = \texttt{cexpr} \mid \texttt{ac} \mid \texttt{abexpr}_1 + \texttt{abexpr}_2 \mid \texttt{abexpr}_1 * \texttt{abexpr}_2$ where ac represents an abstract cost function as defined in Sect. 3.1 and cexpr is a concrete cost expression. The definition above yields linear bounds, however, the extension to infer postconditions in the subsequent section leads to polynomial expressions (of arbitrary degree).[4]

*Example 7 (Abstract Cost Invariant).* Consider the first loop in Example 6 (where *growth* = i) with the following frame and footprint:

```
//@ assignable j; accessible i,t,j,k; cost_footprint k;
```

Using $\mathcal{M}_{\mathsf{instr}}$, the evaluation of the loop guard and the increase of i both have unit cost, so the ACRS is:

$$
\begin{array}{ll}
C(\mathsf{i},\mathsf{t},\mathsf{j},\mathsf{k}) = 1 & \{\mathsf{i} \geq \mathsf{t}\} \\
C(\mathsf{i},\mathsf{t},\mathsf{j},\mathsf{k}) = \mathsf{ac}_{\mathsf{P}}(\mathsf{k}) + 2 + C(\mathsf{i}',\mathsf{t},\_,\mathsf{k}) & \{\mathsf{i}' = \mathsf{i}+1,\ \mathsf{i} < \mathsf{t}\}
\end{array}
$$

The value of the assignable variable j in the recursive call is "forgotten" (item (6) in Def. 3), but this information loss does not affect solvability of the ACRS. We obtain the following ACI: "//@ **cost_invariant** 1 + i * (2 + ac$_\mathsf{P}$(k));".

*Example 8 (Upper Bound Abstract Cost Invariant).* Sometimes an ACI is over-approximating cost, resulting in an *upper bound ACI*. To illustrate this, we add an instruction that creates an array of non-constant size "i" to the program in Example 7 and measure memory consumption instead of instruction count.

```
while (i < t) {
    a = new int[i];
    //@ assignable j;
    //@ accessible i,t,j,a,k;
    //@ cost_footprint k;
    \abstract_statement P;
    i++;
}
```

The resulting ACRS thus accumulates cost "i" at each iteration, plus the memory consumed by the abstract statement:

$$
\begin{array}{ll}
C(\mathsf{i},\mathsf{t},\mathsf{j},\mathsf{k}) = 0, & \{\mathsf{i} \geq \mathsf{t}\} \\
C(\mathsf{i},\mathsf{t},\mathsf{j},\mathsf{k}) = \mathsf{ac}_{\mathsf{P}}(\mathsf{k}) + \mathsf{i} + C(\mathsf{i}',\mathsf{t},\_,\mathsf{k}), & \{\mathsf{i}' = \mathsf{i}+1, \mathsf{i} < \mathsf{t}\}
\end{array}
$$

Now, maximizing the expression $\mathtt{C}_{\mathtt{N}_1} = \mathsf{i}$ under $\{\mathsf{i}' = \mathsf{i}+1, \mathsf{i} < \mathsf{t}\}$ results in $\mathtt{C}_{\mathtt{N}_1}{}^{\mathtt{max}} = \mathsf{t}-1$ and upper bound ACI "//@ **cost_invariant** i * (t − 1 + ac$_\mathsf{P}$(k));".

Let $c_L$ denote the abstract cost of executing a loop $L$ (in analogy to $c_{\mathcal{P}}$ in Def. 2, but considering only loop $L$ rather than the whole program $\mathcal{P}$). We denote by $c_I$ the portion of the cost in $c_L$ up to the execution of iteration $I$.

**Proposition 1.** *Let $L$ be a loop with variables $\overline{x}$ satisfying Def. 4, $\texttt{cinv}(\overline{x})$ its ACI, and $\sigma_I \in \mathbb{Z}^{n_m}$ be the store after performing iteration $I$ of $L$. Then the following holds: (1) $\texttt{cinv}(\overline{x})$ is true on entering the loop; (2) $c_I(\sigma_I) \leq \texttt{cinv}(\sigma_I)$.*

[4] As our approach is based on a recurrences-based framework [39] that works for exponential and logarithmic expressions, the results in this section generalize to these expressions. However, the AE deductive verification system is not able to deal with them automatically at the moment, so we skip these expressions in our account.

### 4.3 From Cost Invariants to Postconditions

To handle programs with nested loops and to prove relational properties it is necessary to infer *cost postconditions* for abstract programs. For nested loops the cost postcondition states the abstract cost after complete execution of the inner loop and it is used to compute the invariant of the outer loop. For relational properties, the cost postconditions of two abstract programs are compared. Cost postconditions for concrete programs are obtained by upper bound solvers (e.g., COSTA [3], CoFloCo [16], AProVE [17]) that compute *max_iter*, an upper bound on the number of iterations that a loop performs. To do so, one relies on ranking functions. We do this as well, but generalize the computation of postconditions to abstract programs. The cost postcondition is obtained by substituting `growth` by `max_iter` in the formula of $\mathtt{cinv}(\overline{x})$ in Def. 6 as follows.

**Definition 7 (Cost Postcondition).** *Let L be a loop, max_iter be an upper bound on the number of iterations of L. Given the ACRS for L in Def. 3, we infer the cost postcondition for L as*

$$post(\overline{x}) = \mathtt{C_B}^{\mathtt{max}} + max\_iter(\overline{x}) \cdot \left(\sum_{j=1}^{n} \mathtt{ac_j}\left(c_{j,1},\ldots,c_{j,h_{cj}}\right) + \sum_{i=1}^{m} \mathtt{C_{N_i}}^{\mathtt{max}}\right)$$

*and generate the annotation "//@ **assert** cost == post($\overline{x}$);".*

To infer the postcondition for a complete abstract program, we take the sum of all *cost postconditions* of its top-level loops plus the cost of the non-iterative fragments. Fig. 1 shows the cost postconditions for our running example obtained by replacing the growth i of the invariant with the bound t on the loop iterations and requiring $\mathtt{t} \geq 0$. The generation of inductive ACIs for nested loops uses the cost postcondition of inner loops to compute the invariants of the outer ones. The following theorem states soundness of cost postconditions:

**Theorem 2.** *Let L be a loop over variables $\overline{x}$ satisfying Def. 4 and $post(\overline{x})$ its cost postcondition. Let $\sigma_L \in \mathbb{Z}^{m_n}$ be the store upon termination of L. Then $c_L(\sigma_L) \leq post(\sigma_L)$.*

## 5 Experimental Evaluation

We implemented a prototype of our approach downloadable from https://tinyurl.com/qae-impl (including required libraries). The archive contains the benchmarks of this section and additional examples as well as build and usage instructions. The prototype is a command-line implementation backed by an existing cost analysis library for (non-abstract) Java bytecode as well as the deductive verification system KeY [2] including the AE framework [37,38]. Our implementation consists of three components: (1) An extension of a cost analyzer (written in Python) to handle abstract Java programs, (2) a conversion tool (written in Java) translating the output of the analyzer to a set of input files for KeY, (3) a bash script orchestrating the whole tool chain, specifically, the interplay between item (1), item (2) and the two libraries. In case of a failed certification attempt, our script offers the choice to open the generated proof in KeY for further debugging. In total, our implementation (excluding the libraries) consists of 1,802 lines of Python, 703 lines of Java, and 389 lines of bash code (without blank lines and comments).

To assess effectiveness and efficiency of our approach, we used our QAE implementation to analyze seven typical code optimization rules using cost models $\mathcal{M}_{\mathsf{instr}}$ (rows "1∗"–"6∗" in Table 1) and $\mathcal{M}_{\mathsf{heap}}$ (rows "7∗"). While $\mathcal{M}_{\mathsf{instr}}$ counts the number of instructions, $\mathcal{M}_{\mathsf{heap}}$ measures heap consumption. The first column identifies the benchmark ("a" refers to the original program, "b" to the transformed one), the second **P** refers to the kind of proven cost result (asymptotic "a", exact "e", upper "u"), column three shows the inferred growth function for each loop in the program (separated by "," if there are two or more loops), in the fourth column we list the cost postcondition obtained by the analysis (expressions indicating the number of loop iterations are highlighted), and columns five to eight display performance metrics. Time $t_{\mathtt{cost}}$, given in milliseconds, is the time needed to perform the cost analysis. The proof generation time $t_{\mathtt{proof}}$ is given in seconds. We also display the time $t_{\mathtt{check}}$ needed for checking integrity of an already generated proof certificate. Finally, $s_{\mathtt{proof}}$ is the size of the generated KeY proof in terms of number of proof steps. Even though the time needed for certification is significantly higher than for cost analysis (which is to be expected), each analysis can be performed within one minute. The time to *check* a proof certificate amounts to approximately one fourth to one third of the time needed to *generate* it. We stress that all analyses are *fully automatic*.

We briefly describe the nature of each experiment: **1** is a *loop unrolling* transformation duplicating the body of a loop: each copy of the body is put inside an **if** -statement conditioned by the loop guard. Here, we had to switch to *asymptotic* cost invariants: The cost analyzer over-approximates the number of iterations of the unrolled loop, since there are different possible control flows in the body. This was automatically detected by the certifier which failed to find a proof when exact cost invariants are conjectured and succeeds with asymptotic ones. **2** is the *CodeMotion* example from Sect. 2. The result reflects the cost *decrease* in the sense that less instructions need to be executed by the transformed program. **3** implements a *LoopTiling* optimization at compiler level in which a single loop with $n \cdot m$ iterations is transformed into two nested loops, an outer one looping until $n$ and an inner one until $m$. Since our cost analyzer only handles linear size expressions, the first program is written using an auxiliary parameter $t$ that is then instantiated to value $n \cdot m$. **4** is a *SplitLoop* transformation splitting a loop with two independent parts into two separate loops. We prove that this transformation does not affect the cost up to a constant factor. **5** is an optimization combining *two loops* with the same body structure into one loop. **6** is a *three loops* example, one nested and one simple. The optimization combines the bodies of the outer loop in the nested structure and the simple loop. **7** is an *array* optimization, where an array declaration is moved in front of a loop, initializing it with an auxiliary parameter that is the sum of all the initial sizes.

| | P | Cost analysis results | | $t_{\text{cost}}$ [ms] | $t_{\text{proof}}$ [s] | $t_{\text{check}}$ [s] | $s_{\text{proof}}$ #nodes |
|---|---|---|---|---|---|---|---|
| | | **Growth** | **Postcondition** | | | | |
| **1a** | a | $i$ | $t{\cdot}\mathsf{ac}_{\mathsf{P}}(x)$ | 45.0 | 12.9 | 4.3 | 1,784 |
| **1b** | a | $i$ | $t{\cdot}\mathsf{ac}_{\mathsf{P}}(x)$ | 53.4 | 23.8 | 5.0 | 3,472 |
| **2a** | e | $i$ | $2{+}t{\cdot}(7+\mathsf{ac}_{\mathsf{P}}(t,w)+\mathsf{ac}_{\mathsf{Q}}(t,z))$ | 50.0 | 23.3 | 5.7 | 3,692 |
| **2b** | e | $i$ | $3+\mathsf{ac}_{\mathsf{P}}(t,w){+}t{\cdot}(6+\mathsf{ac}_{\mathsf{Q}}(t,z))$ | 42.0 | 19.7 | 5.7 | 3,243 |
| **3a** | e | $i$ | $2{+}t{\cdot}(6+\mathsf{ac}_{\mathsf{P}}(k))$ | 49.1 | 18.7 | 5.1 | 2,821 |
| **3b** | e | $i\ ,\ j$ | $6{+}n\cdot m{\cdot}(6+\mathsf{ac}_{\mathsf{P}}(k))$ | 49.5 | 23.3 | 5.7 | 3,794 |
| **4a** | e | $i+1$ | $2{+}(l+1){\cdot}(7+\mathsf{ac}_{\mathsf{Q1}}(t,w)+\mathsf{ac}_{\mathsf{Q2}}(t,z))$ | 49.5 | 23.8 | 5.7 | 3,933 |
| **4b** | e | $i+1\ ,\ i+1$ | $2{+}(l+1){\cdot}(12+\mathsf{ac}_{\mathsf{Q1}}(t,w)+\mathsf{ac}_{\mathsf{Q2}}(t,z))$ | 48.5 | 29.4 | 7.3 | 5,137 |
| **5a** | e | $i\ ,\ j$ | $2{+}n{\cdot}(6+\mathsf{ac}_{\mathsf{P}}(y)){+}\mathrm{m}{\cdot}(6+\mathsf{ac}_{\mathsf{P}}(y))$ | 55.1 | 25.3 | 7.1 | 4,795 |
| **5b** | e | $i$ | $2{+}(n+m){\cdot}(8+\mathsf{ac}_{\mathsf{P}}(y))$ | 48.2 | 14.1 | 4.7 | 2,492 |
| **6a** | e | $k\ ,\ j\ ,\ n-i$ | $6{+}n{\cdot}(m{\cdot}(6+\mathsf{ac}_{\mathsf{P}}(y)){+}n{\cdot}(5+\mathsf{ac}_{\mathsf{Q}}(y))$ | 49.8 | 32.0 | 8.1 | 7,078 |
| **6b** | e | $k\ ,\ j$ | $7{+}n{\cdot}(m{\cdot}(6+\mathsf{ac}_{\mathsf{P}}(y))+\mathsf{ac}_{\mathsf{Q}}(y))$ | 49.6 | 24.9 | 6.4 | 4,995 |
| **7a** | u | $i-1$ | $(t-1){\cdot}(4\cdot(t-1)+\mathsf{ac}_{\mathsf{P}}(y))$ | 51.2 | 15.6 | 5.3 | 2,578 |
| **7b** | u | $i-1$ | $4\cdot m{+}(t-1){\cdot}\mathsf{ac}_{\mathsf{P}}(y)$ | 43.3 | 13.0 | 4.2 | 1,793 |

Table 1: Results of the experiments.

## 6 Related Work

The present paper builds on the original AE framework [37,38], which we extend to *Quantitative* AE. At the moment no other approach or tool is able to analyze and certify the cost of schematic programs, specifically relational properties, so a direct comparison is impossible.

*Cost Analysis.* There are many resource analysis tools, including: [20], based on introducing counters and inferring loop invariants; [23], based on an analysis over the depth of functional programs formalized by means of type systems. Approaches that bound the number of execution steps include [19,29], working at the level of compilers. Systems such as APROVE [17] analyze the complexity of JAVA programs by transforming them to integer transition systems; COSTA [3] and COFLOCO [16] are based on the generation of cost recurrence equations from which upper bounds can be inferred. That is also the basis of the approach we pursue to infer abstract upper bounds in Sect. 4.1, hence our technique can be viewed as a generalization of these systems. Approaches based on type systems could also be generalized to work on abstract programs by introducing abstract cost as in Sect. 4.1.

For our work it is crucial to use ranking functions to infer growth of cost invariants. Ranking functions were used to generate bounds on the number of loop iterations in several systems, but none used them to define growth: [10] obtain runtime complexity bounds via symbolic representation from ranking functions, likewise PUBS [3], LOOPUS [40], and ABC [8]. PUBS analyses all loop transitions at once, LOOPUS uses an iterative procedure where bounds are propagated from inner to outer loops, ABC deals with nested, but not sequential loops. In our work, when inferring upper bounds, we solve all transitions at once and handle nested as well as sequential loops.

*Certification.* Several general-purpose deductive software verification [21] tools exist, including VeryFast [34], Why [15], Dafny [28], KIV [33], and KeY [2]. We use KeY, the currently only system to implement AE. *Interactive* proof assistants like Isabelle [31] or Coq [7] also support more or less expressive abstract program fragments, but lack full automation. There are dedicated approaches involving schematic programs for *specific* contexts, like regression verification [18], compilation [22, 26, 30] or derived symbolic execution rules [12].

Regarding the combination of deductive verification and cost analysis, the closest approach to ours is the integration of COSTA and KeY [4] which was realized for concrete, not abstract programs. They verify upper bounds on the cost of concrete programs by decomposing them into ranking functions and size relations which are then verified separately. Here we use the novel concept of cost invariant that allows verification of quantitative properties without decomposition. Paper [4] deals only with the global number of iterations as is common in worst-case cost analysis. Our cost invariants are designed to be inductive and propagate cost through all loop iterations. Radiček et al. [32] devise a formal framework for analyzing the relative cost of different programs (or the same program with different inputs). Compared to our approach, they target purely functional programs extended with monads representing cost, while we work with an industrial programming language. Moreover, we generally reason about the cost of *transformations*, not of a transformation applied to one *particular* program.

## 7 Conclusion and Future Work

We presented the first approach to analyze the cost of schematic programs with placeholders. We can infer and verify cost bounds for a potentially infinite class of programs once and for all. In particular, for the first time, it is possible to analyze and prove changes in efficiency caused by program transformations—for all input programs. Our approach supports exact and asymptotic cost and a configurable cost model. We implemented a tool chain based on a cost analyzer and a program verifier which analyzes and formally certifies abstract cost bounds in a fully automated manner. Certification is essential, because only the verifier can determine whether the bounds inferred by the cost analyzer are exact.

Our work required the new concept of an (abstract) cost invariant. This is interesting in itself, because (i) it renders the analysis of nested loops modular and (ii) provides an interface to backends (such as verifiers) that characterizes the cost of code in iterations.

Obvious future work involves extending the analyzed target language. Cost analysis and deductive verification (including AE) are already possible for a large Java fragment [3, 37]. More interesting—and more challenging—is the analysis of program transformations that parallelize code. The extension to larger classes of cost functions, such as logarithmic or exponential, could be realized by integrating non-linear SMT solvers into the tool chain.

*Acknowledgments.* This work was funded partially by the Spanish MCIU, AEI and FEDER(EU) project RTI2018-094403-B-C31, by the CM project S2018/TCS-4314 co-funded by EIE Funds of the EU and by the UCM CT42/18-CT43/18 grant.
